# Vertically-Oriented WS_2_ Nanosheets with a Few Layers and Its Raman Enhancements

**DOI:** 10.3390/nano10091847

**Published:** 2020-09-16

**Authors:** Yukyung Shin, Jayeong Kim, Yujin Jang, Eunji Ko, Nam-Suk Lee, Seokhyun Yoon, Myung Hwa Kim

**Affiliations:** 1 Department of Chemistry & Nanoscience, Ewha Womans University, Seoul 03760, Korea; shinyk@ewhain.net; 2 Department of Physics, Ewha Womans University, Seoul 03760, Korea; jayeongkim@ewhain.net (J.K.); yujin97@ewhain.net (Y.J.); ellenko94@ewhain.net (E.K.); 3 National Institute for Nanomaterials Technology (NINT), Pohang University of Science and Technology (POSTECH), Pohang 37673, Korea; nslee@postech.ac.kr

**Keywords:** tungsten disulfide, nanosheet, chemical vapor deposition, surface-enhanced Raman scattering

## Abstract

Vertically-oriented two-dimensional (2D) tungsten disulfide (WS_2_) nanosheets were successfully grown on a Si substrate at a temperature range between and 550 °C via the direct chemical reaction between WCl_6_ and S in the gas phase. The growth process was carefully optimized by adjusting temperature, the locations of reactants and substrate, and carrier gas flow. Additionally, vertically-oriented 2D WS_2_ nanosheets with a few layers were tested as a surface-enhanced Raman scattering substrate for detecting rhodamine 6G (R6G) molecules where enhancement occurs from chemical enhancement by charge transfer transition from semiconductor). Raman spectra of R6G molecules adsorbed on vertically-oriented 2D WS_2_ nanosheets exhibited strong Raman enhancement effects up to 9.2 times greater than that on the exfoliated WS_2_ monolayer flake sample. From our results, we suggest that the WS_2_ nanosheets can be an effective surface-enhanced Raman scattering substrate for detecting target molecules.

## 1. Introduction

Considerable attention has been devoted to two-dimensional (2D) materials as promising functional materials, in particular to transition metal dichalcogenides (TMDCs) [1,2,3,4], due to rapid advances in synthesis, transfer, spectroscopic detection, and manipulation. Since 2D TMDCs have unique physicochemical properties such as high mobility, large surface area, and significant catalytic activities, they can be effectively used for efficient light harvesting, sensitive photo-detection, and catalytic conversion systems [5,6,7,8]. Among 2D TMDCs, particularly, tungsten disulfide (WS_2_), with direct optical band gaps of 1.35 and 2.05 eV for bulk and monolayer structure, is of great interest due to its particular semiconducting behavior, intrinsic electrical conductivity, and electrocatalytic property when the number of layers is lower [9,10,11]. Recent work shows that 2D WS_2_ nanostructures could exhibit the enhancement of Raman signals for adsorbed molecules as a function of the number of layers [12]. Although a variety of synthetic methodologies were thus suggested to grow single and few-layer nanosheets of the high-quality 2D WS_2_, the synthesis and characterization of vertically-oriented 2D WS_2_ nanosheets have rarely been reported [13,14,15,16].

In this work, we described a novel growth process and its characterizations of vertically-oriented 2D WS_2_ nanosheets using chemical vapor deposition (CVD). Although Li et al. previously reported MoS_2_ and WS_2_ inorganic fullerene-like nanostructures and nanoflowers with a similar growth method using atmospheric pressure chemical vapor deposition (APCVD) [17], our growth process makes it possible to obtain vertically-oriented 2D WS_2_ nanosheets with a few layers on a Si substrate by carefully controlling the gas flow and the different location of the substrate at a much lower temperature. Thus, this synthetic methodology could offer an efficient growth process for 2D materials with high density. To utilize our structure, we performed surface-enhanced Raman scattering (SERS) experiments for rhodamine 6G (R6G) molecules adsorbed on vertically-oriented layers of 2D WS_2_ nanosheets. It is well known that SERS could enhance inherently weak Raman scattering cross-sections by many orders of magnitude using specially-prepared substrates on which samples to be measured are adsorbed [18,19,20,21]. Most of SERS substrates consist of noble metals using surface plasmon resonance in the visible range; however, semiconducting substrates are also being used for decent enhancement, whose enhancement mechanism is mainly associated with charge transfer between the substrate and the adsorbate molecules [22,23,24]. Recently, 2D materials, including TMDC materials are also being used as SERS substrates [25,26,27].

## 2. Materials and Methods

**Figure 1:**
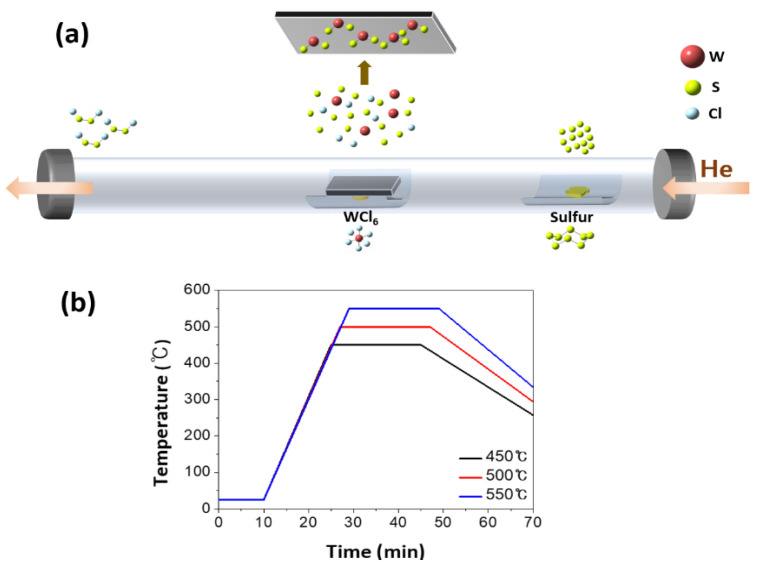
(**a**) Scheme of the chemical deposition process of the WS_2_ nanosheets and (**b**) temperature profile during the growth process.

To grow 2D WS_2_ nanosheets, tungsten hexachloride (WCl_6_, Sigma-Aldrich, Seoul, Korea) was placed in the quartz boat at the center of the quartz tube of 60 cm length with a 2.5-inch diameter in the furnace, as illustrated in Figure 1 [28]. Another quartz boat containing sulfur powder (Sigma-Aldrich, 99.98%, Seoul, Korea) was then placed at 15 cm upstream from the quartz boat with WCl_6_, which was near the outside edge of the heat zone in the furnace. The Si(001) substrate at the growth region was placed face-down above the quartz boat with WCl_6_. The gap distance between WCl_6_ and the Si substrate was approximately 10 mm. Before starting the growth process, the quartz tube was flushed with the carrier gas helium (He, 99.999%, Dong-A Industrial Gas, Seoul, Korea) for 10 min at 400 standard cubic centimeter per minute (sccm). The furnace was gradually heated up to 450~550 °C at the ramping speed of 26 °C per minute and maintained at the growth temperature for 20 min. During the growth process, the temperature at the location of the sulfur powder was kept between 180 and 200 °C. For the whole process, helium (He) was continuously flowed at the flow rate of 10 sccm. After finishing the growth process, the furnace (Lindberg/Blue M, Thermo Fisher Scientific, Waltham, MA, USA) was turned off and gradually cooled down to room temperature.

The characteristics of 2D WS_2_ nanosheets on a Si substrate were analyzed by scanning electron microscopy (FE-SEM, JEOL JSM-6700F, Tokyo, Japan), high-resolution transmission electron microscopy (HR-TEM, with a probe Cs-corrector, JEM-2100F, Tokyo, Japan), X-ray diffraction (XRD, Rigaku diffractometer, Tokyo, Japan with Ni filtered Cu-Kα radiation, λ = 0.15418 nm), X-ray photoelectron spectroscopy (XPS with a Theta probe AR system and X-ray source with monochromated Al Kα, hν = 1486.6 eV, JEOL, Tokyo, Japan), and Raman spectroscopy (Horiba, Kyoto, Japan). The atomic ratios of W/S in the form of nanosheets were carefully measured by energy-dispersive X-ray spectroscopy (EDX).

For Raman measurements, 5 × 10^−4^ M R6G solution was prepared by adding 500 µL of ethanol to 500 µL of 10^−3^ M R6G aqueous solution for effective drying. We drop-casted 12.5 µL of R6G solution on 2D WS_2_ nanosheets and exfoliated WS_2_ monolayer flake samples and waited for natural drying. Raman spectra of R6G molecules adsorbed on WS_2_ samples were measured by using a McPherson 207 spectrometer equipped with a nitrogen-cooled charge-coupled-device (CCD) array detector at room temperature. The samples were excited with a 532 nm (2.33 eV) diode-pumped solid state (DPSS) laser, focused to ~1 μm diameter spot using a microscope objective (100×). The excitation power was less than 0.05 mW to avoid laser heating and the exposure time was 60 s.

To estimate local electric field response distribution near the WS_2_ surface, finite-difference time-domain (FDTD) calculations (Lumerical Inc., Vancouver, Canada) were carried out. As the size of each bundle of nanosheets is a few μm and the size of the laser beam used in the Raman experiment is about 1 μm, we built the flower-like structure by making a board that was 1 × 0.01 × 0.4 μm and rotating it around a vertical (z) axis so that it has 16 leaves. A plane-wave source was used and the boundary conditions for the periodic structure were configured: anti-symmetric for × boundaries, symmetric for y boundaries, and perfectly matched layer (PML) for z boundaries. Due to the thickness of single layer R6G molecules, we set the maximum mesh size over the nanoflower structure as 0.3–1 nm.

## 3. Results and Discussion

**Figure 2:**
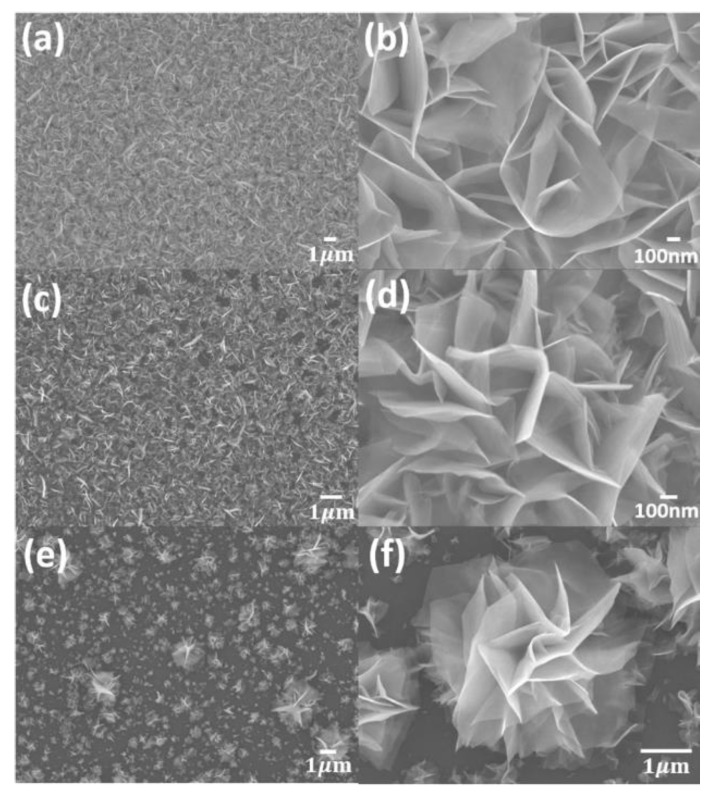
SEM images of vertically-oriented WS_2_ nanosheets grown on a Si substrate at (**a**,**b**) 450, (**c**,**d**) 500, and (**e**,**f**) 550 °C.

Figure 2 shows SEM images of 2D WS_2_ nanosheets grown at 450, 500, and 550 °C on a Si substrate. In SEM images, the morphology of the thin-layered WS_2_ nanostructures clearly represents a distinct flower-like nanostructure by standing on the vertical direction against the substrate. The density of vertically-oriented 2D WS_2_ nanostructures gradually decreased likely because 2D WS_2_ nanosheets with larger sizes formed due to the faster growth rate at higher temperature. In Figure 2b,d,f, the high magnification SEM images indicate that the lateral size of the 2D WS_2_ nanosheets is approximately 2 μm and their thickness is less than 10 nm. This kind of asymmetric crystal growth in the out-of-plane might be responsible for the weak interlayer coupling between WS_2_ crystal structures. Particularly, the favorable vertical orientation of the 2D WS_2_ nanosheets might be attributed to the compression and extrusion between WS_2_ island layers initially formed on the Si substrate [16]. In addition, the face-down position of the Si substrate with respect to the WCl_6_ source would be beneficial to obtaining a high flux of supersaturated WS_2_ vapors into the Si substrate, resulting in the vertical growth of WS_2_ nanosheets [16,27].

**Figure 3:**
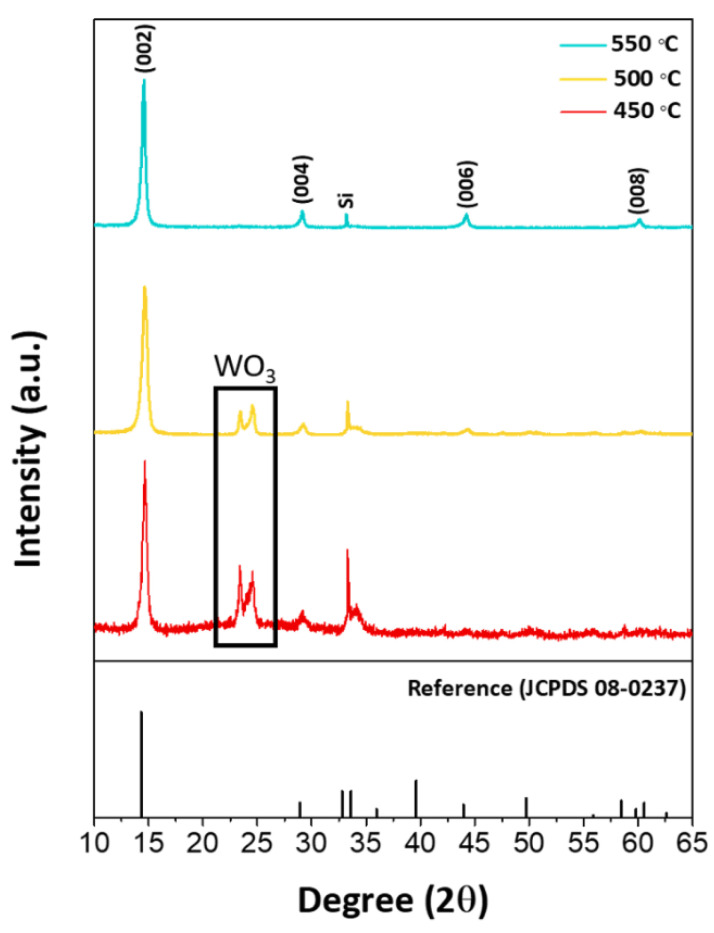
XRD patterns of vertically oriented WS_2_ nanosheets grown on a Si substrate at 450, 500 and 550 °C.

Figure 3 shows the XRD pattern of the 2D WS_2_ nanosheets grown at different temperatures, indicating that there are only four prominent peaks at 14.6°, 29.1°, 44.2°, and 60.2°, which can be attributed to (002), (004), (006), and (008) planes of the hexagonal phase (JCPDS card No: 08-0237), respectively [29]. Whereas the XRD pattern at 550 °C illustrates the pure hexagonal WS_2_ crystalline phase, the XRD peaks between 23° and 25° at 450 °C and 500 °C are associated with WO_3_ impurity. Interestingly, the distinct appearance of only (00*l*) reflections represents the characteristic of the specific orientation of the 2D WS_2_ crystal structure along the *c* axis direction. Based on the peak at 14.6° assigned as the (002) plane, the lattice constant of *c* axis is estimated as 12.22 Å. Additionally the intense sharp peaks at 550 °C confirm the highly crystalline nature of 2D WS_2_ layered structures.

**Figure 4:**
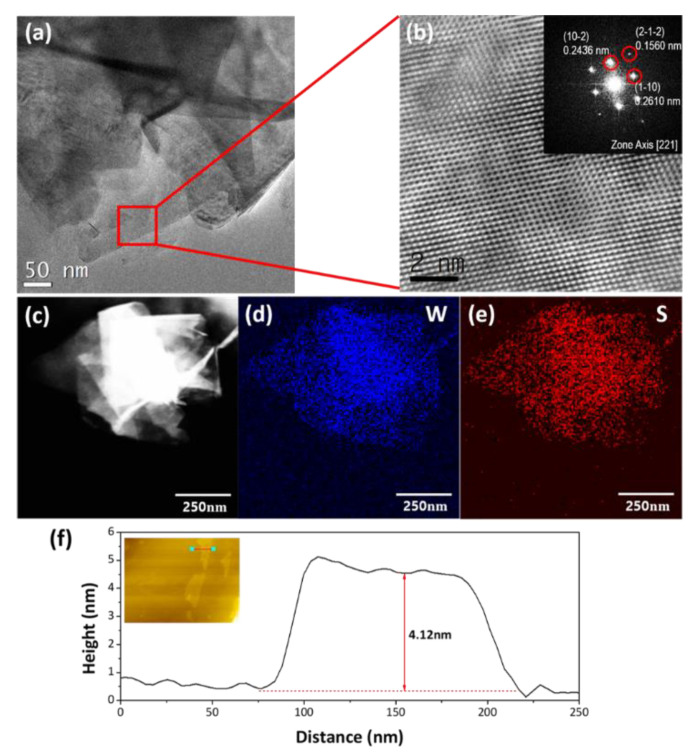
(**a**) Low-magnification TEM image of WS_2_ nanosheets at 550 °C, (**b**) high-resolution TEM image and the fast Fourier transform (FFT) pattern of WS_2_ nanosheets(inset) at 550 °C, (**c**–**e**) EDS elemental mapping analysis of W and S atoms in WS_2_ nanosheets, (**f**) the height profile of WS_2_ nanosheets grown at 550 °C by atomic force microscopy (AFM) line scan.

In the TEM measurement, the formation of different numbers of layered WS_2_ nanosheets is clearly identified by the relative difference in brightness with distinct edges as shown in Figure 4a. The fast Fourier transform (FFT) image in Figure 4b inset also confirms the existence of specific crystalline planes, corresponding to the WS_2_ crystal structures, indicating the highly single-crystalline nature of the nanosheet. Energy-dispersive X-ray spectroscopy (EDS) elemental mapping analysis from the Z-contrast high-angle annular dark-field scanning transmission electron microscopy (HAADF-STEM) image exhibits that W and S atoms are homogeneously distributed to the entire nanosheet, resulting in the almost stoichiometric atomic ratio of 1:1.97.

The atomic force microscopy (AFM) measurement in Figure 4f depicts that the height of a single WS_2_ nanosheet is about 4.12 nm, which corresponds to about five layers of the WS_2_ nanosheets.

**Figure 5:**
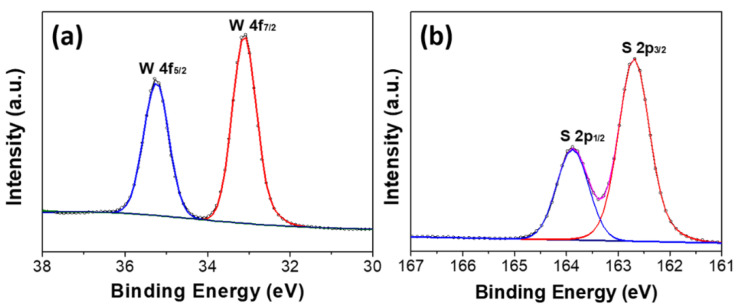
X-ray photoelectron spectroscopy (XPS) spectra of two-dimensional (2D) WS_2_ nanosheets at 550 °C and (**a**) W 4f and (**b**) S 2p regions.

Figure 5 represents the XPS spectra for the WS_2_ nanosheets, which could provide information regarding the surface composition and the chemical states of the elements in nanostructures. XPS spectra of WS_2_ nanosheets grown at 550 °C indicates that while the binding energies of 33.08 and 35.0 eV are associated with the W 4f_7/2_ and W 4f_5/2_ core level peaks, two main peaks at 162.7 and 163.9 eV correspond to S 2p_3/2_ and 2p_1/2_ of the divalent ion (S^2−^) state, respectively [30]. These features are closely consistent with the XPS spectra for the exfoliated bulk WS_2_ structure.

In our study, the growth of the WS_2_ nanosheets could be interpreted by the direct chemical reaction between WCl_6_ and S atoms in the gas phase on a Si substrate under the flow of the carrier gas (He) as given in Equation (1) [17]:

WCl_6_ + 8S ⟶ WS_2_ + 3S_2_Cl_2_ (1)

It is suggested that the reaction between WCl_6_ and S atoms initially forms tiny WS_2_ nuclei at the growth temperature. The continuous feeding of WS_2_ into the nucleation sites can then lead to the two-dimensional layered structure of WS_2_ as a favorable form. In addition, the use of excessive S powder is beneficial for completely changing the chlorides to WS_2_. Generally, since the reaction in Equation (1) is highly exothermic at elevated temperatures, it leads to a rapid reaction progression with a high degree of supersaturation of the vapors and, hence, to fast nucleation. Thus, we expected that numerous nuclei of WS_2_ could be initially formed in the vapor phase at the near heating zone. It is likely that some nuclei grow larger relative to others and subsequently precipitate on the substrates, which are placed at the upper position from the WCl_6_ precursors. The deposited WS_2_ nanostructures then play a crucial role as the efficient growth template and promote the subsequent vertical growth of WS_2_ nanosheets due to the high strain energy by the compression between WS_2_ island layers as previously proposed [16]. During the growth process at high temperature, additionally, the gravity force might affect the vertical orientation of layered structures with relatively large radii of curvature and thin walls from deposited WS_2_ nuclei on a Si substrate, resulting in flower-like nanostructures. Although the whole reaction system was tightly sealed and protected by a flow of He, traces of oxygen could cause the oxidation of the products. As shown in Figure 3, the minor product, WO_3_, was inevitable at the growth temperature of 450 and 500 °C, whereas it was completely absent at 550 °C. Therefore, this suggested that the growth temperature plays a critical role in efficiently obtaining the WS_2_ nanosheets with high crystallinity and high density on a Si substrate due to different thermodynamics and kinetics for the growth behavior.

**Figure 6:**
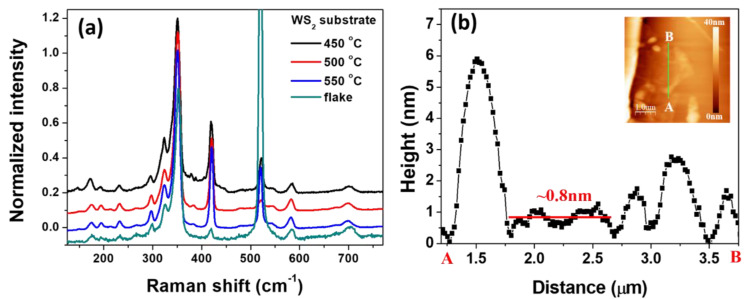
(**a**) Raman spectra of 2D WS_2_ nanosheets substrates and monolayer WS_2_ flake substrate. Spectra are offset for clarity. (**b**) AFM topography line profile results of a WS_2_ flake sample, showing the WS_2_ flake sample is monolayer WS_2_.

Figure 6a represents the Raman spectra of 2D WS_2_ nanosheets substrates grown at different growth temperatures and an exfoliated flake sample. All the nanosheet samples were of high quality as they all showed characteristic WS_2_ phonon peaks at 532 nm excitation as an exfoliated flake sample. Figure 6b shows the topography and line profile of the exfoliated flake sample from which the flake is seen to be monolayer-thick. Note that the intensity of A_1g_ (Γ) peak at ~420 cm^−1^ in nanosheet samples is much stronger. Suggesting that the nanosheet samples were much thicker than the exfoliated flake sample, which is also confirmed from Figure 1 where the thicknesses of the nanosheets are measured as ~10 nm.

**Figure 7:**
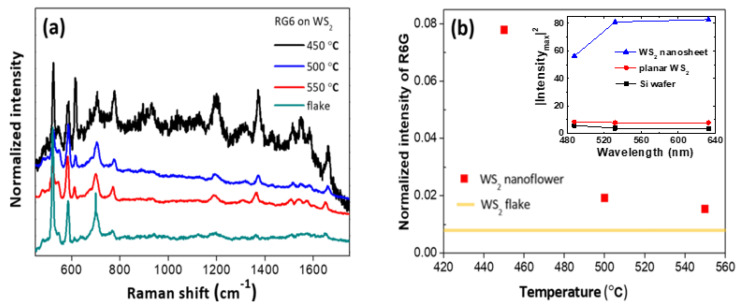
(**a**) Raman spectra of R6G molecules adsorbed on WS_2_ nanosheets substrates grown at 450, 500, and 550 °C, and an exfoliated WS_2_ monolayer flake sample. Spectra are offset for clarity. Intensities are normalized to the intensity of WS_2_ E^1^_2g_ mode. (**b**) Normalized (to WS_2_ E^1^_2g_ mode) intensity of R6G 1361 cm^−1^ mode. Filled squares represent the normalized intensities observed from WS_2_ nanoflower (nanosheet) samples, and the straight line represents the normalized intensity observed from an exfoliated WS_2_ monolayer flake. Inset: Finite-difference time-domain (FDTD) calculation results of scattered electric field intensity squared as a function of incident wavelength. Note that scattered intensities from both WS_2_ samples are larger than that from Si wafer confirming that it is from surface-enhanced Raman scattering (SERS) effects.

Figure 7 shows the Raman spectra of R6G molecules deposited on the 2D WS_2_ nanosheets grown at various temperatures and an exfoliated WS_2_ monolayer flake. In Figure 7a, vibration modes of adsorbed R6G molecules are clearly shown at 612, 770, 1181, 1303, 1361, 1500, 1570, and 1650 cm^−1^ from all the 2D WS_2_ substrates. The strongest intensity of R6G molecular vibrations was observed in the 2D WS_2_ nanosheets grown at 450 °C and the intensity was weaker for nanosheets with higher growth temperatures. Since we did not observe any R6G Raman signal from solution with R6G concentration of 5 × 10^−4^ M and we only observed R6G vibration peaks when we drop-casted the R6G solution on WS_2_ substrates, the observation of R6G Raman responses is a direct result of surface-enhanced Raman scattering (SERS).

Since WS_2_ is a semiconductor, its surface plasmon energy is far from the visible range and the enhancement is entirely due to chemical, or charge transfer, enhancement. In our case, with visible (2.33 eV) excitation, charge transfer can only occur from the valance band (VB) of WS_2_ to the lowest unoccupied molecular orbital (LUMO) level of R6G. The largest Raman enhancement thus occurs when the energy difference between VB and LUMO matches the excitation energy of the incident laser [31,32]. The samples grown at different temperatures have different thicknesses and different impurity contents, leading to different positions of each energy level. As a result, the relevant energy difference between VB and LUMO is different sample by sample and it is likely that the 450 °C sample has an energy difference close to the laser excitation energy.

Another factor that can affect the enhancement is the difference in the surface area among samples, which is directly related to the amount of charge transfer between the substrate and the sample. However, this was less likely in our case because the diameter of the focused laser beam was about 1 μm, which is larger, or at least comparable, to each flower structure, so the difference between the surface area from different samples was not significant. Although the intensity of R6G molecules from vertically-oriented 2D WS_2_ nanosheets grown at 550 °C was the lowest among the three samples, it was still more than twice as strong as the measured intensity from an exfoliated flake sample. The Raman response in vertically-oriented WS_2_ nanosheets was estimated to be up to 9.2 times greater than in the monolayer flake sample as shown in Figure 7b. This can be explained by the difference in surface area, which is much larger in nanosheets. As can be seen in the Figure 7b inset, the FDTD calculation also confirms that the scattered intensity from a nanosheet sample was always larger than that from a planar sample. The difference depends on the incident light wavelength and the intensity was ~4.6 times larger in a nanosheet sample than that in a planar sample for 532 nm excitation, which is consistent with the experimental results. Consequently, this suggests that vertically-oriented 2D WS_2_ nanosheets are much more efficient than exfoliated flakes as SERS substrates. Note that since the main cause of Raman enhancement in our case was not surface plasmon resonance but the photo-induced charge transfer between molecules and substrates, the FDTD results should be taken as an indicator as to how structure/morphology affect the local electric field response in our case.

## 4. Conclusions

We successfully introduced a novel synthesis of vertically-oriented 2D WS_2_ nanosheets with a few layers in high density via chemical vapor deposition using WCl_6_ as the W precursor and sulfur by carefully controlling the gas flow and the location of the substrate at low temperature. Raman spectra of R6G molecules adsorbed on vertically-oriented 2D WS_2_ nanosheets exhibited strong Raman enhancement effects compared to the monolayer flake of 2D WS_2_. This suggested that the charge transfer transition from semiconductor substrates to molecular LUMO may be attributed to an efficient or a dominant process for attaining a large Raman enhancement.
